# Co(II)-Mediated Catalytic Chain Transfer Polymerization (CCTP) Carried Out Under Flow Reaction Conditions and Introducing a New Method for Online GPC Monitoring

**DOI:** 10.1021/acspolymersau.5c00020

**Published:** 2025-05-29

**Authors:** Yanpu Yao, Xiaofan Yang, Cansu Aydogan, James Town, William Pointer, David M. Haddleton

**Affiliations:** † Department of Chemistry, 2707 University of Warwick, Coventry CV4 7AL, United Kingdom; ‡ Polymer Characterization Research Technology Platform, University of Warwick, Coventry CV4 7AL, United Kingdom

**Keywords:** flow chemistry, CCTP, online GPC, online monitoring, CoBF

## Abstract

We report an investigation into the thermally induced catalytic chain transfer polymerization (CCTP) using bis­[(difluoroboryl)­dimethylglyoximato] cobalt­(II) (CoBF) as a chain transfer agent in three different flow reactors: (1) a cascade of continuous stirred-tank reactors (CSTRs), (2) a simple tubular flow reactor, and (3) a Corning Advanced Flow Reactor (AFR). Systematic variations in monomer type, temperature, and stirring rate were employed to investigate their effects on the polymerization process. In the CSTR cascade, higher polymerization rates and conversions were observed without compromising reaction control. Comparative analyses between the flow systems and conventional batch reactions were performed to assess the performance of CoBF under these different reaction conditions. All reactor designs proved successful in carrying out CCTP, and this chemistry is well-suited to continuous production under different flow conditions. The applicability of the reaction system was further verified with successful CCTP of glycidyl methacrylate, and the reproducibility was confirmed by using online continuous GPC.

## Graphical abstract

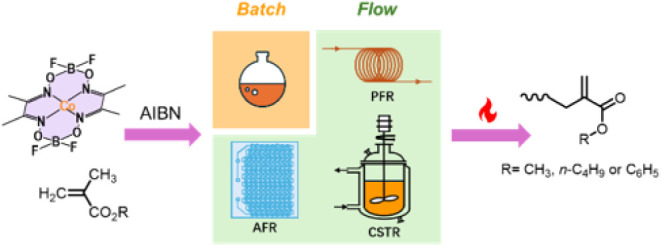

## Introduction

Flow chemistry is being increasingly explored as a technology with intrinsic features that facilitate and provide reproducible and sustainable access to a broad range of chemical processes carried out in continuous processes.^1–7^ This approach is of interest in both academic research and chemical production due to perceived advantages, including enhanced efficiency, reproducibility, and improved safety for exothermic polymerizations, high pressures, and high temperatures.^8–20^ Flow chemistry has previously been applied to various polymerization mechanisms, including anionic, cationic, radical, and ring-opening polymerization, often enabling the preparation of polymers with well-defined molecular weights and structures, and indeed, it is noted that the most high-volume thermoplastic, polyethene, is largely manufactured using a flow reactor design, which achieves moderate conversions and allows for recycling of unreacted monomers in a continuous process.^21–34^ Indeed, one of the advantages of flow chemistry is that conversions need not reach 100% with volatile reagents, as it is quite easy to devolatilize these monomers and solvents and recycle them back into the process. This, for example, was demonstrated by ICI in a continuous GTP process for the polymerization of MMA in an extruder under adiabatic conditions.^35^ It is noted that one of the differences between carrying out polymerizations in flow, as compared to small molecule chemistry, is the increase in viscosity as polymers are formed, especially as molecular weight increases, which has an effect on mixing and thus potentially the reaction efficiency. Consequently, polymerizations are often stopped at less than 100% conversion, followed by devolatilization of volatiles, which are subsequently fed back into the reagent stream.

In flow reactors, all reagents are usually continuously fed to the reactor from one or more reagent streams prior to mixing and then passed through the reaction stage/tube under appropriate conditions, with the product leaving the system continuously. The internal flow dynamics within the reactors vary depending on the reactor’s structural design, resulting in different mixing and, thus, reaction homogeneity. The ideal model of typical flow reactors can be considered as two extreme types: plug flow and flow through a perfectly mixed vessel.^36–40^ In plug flow reactors (PFRs), reactants are typically fed through a simple tubular reactor, with diameters ranging from less than a few millimeters to several centimeters, depending on factors such as cost, residence time, product volume, and heat transfer requirements. In an ideal PFR, the fluid is modeled as a series of coherent ″plugs″ each with a uniform composition, traveling in the direction of the flow. These plugs differ in composition along the reactor’s length, enabling the continuous conversion of reactants into products as the fluid progresses through the reactor. However, in practice, most tubular reactors exhibit laminar flow, where the fluid can be considered to move in thin, parallel layers. Axial dispersion across these layers can lead to a nonideal residence time distribution, potentially affecting the efficiency of the chemical reactions by causing incomplete mixing, which can lead to broadening of the dispersity and, indeed, increasing the product heterogeneity in all respects.^41–45^ Conversely, in a continuous stirred-tank reactor (CSTR), reagents are continuously introduced into the reactor, either by gravity or, more usually, using appropriate pumps. Like a batch reactor, a CSTR employs an agitator to disperse the reactants upon entry to the reaction area, aiming to maintain a uniform composition throughout the reactor. The behavior of a CSTR is often approximated by the ideal CSTR model, which assumes perfect mixing. However, in real-world applications, CSTRs rarely achieve ideal behavior. Nonideal parameters include reactor dead space or short-circuiting, which often arise within the reactor. These issues lead to deviations from the ideal residence time distribution, impacting the efficiency and effectiveness of the reactions.^46^ One approach to overcome this nonidealization is the tanks-in-series (TIS) model, where multiple CSTRs are connected in series to simulate a narrower, more ideal residence time distribution. This is often referred to as a CSTR cascade.^47–50^ However, this method can increase capital costs and space requirements, which can be challenging to implement, particularly in laboratory settings. We also report the use of a Corning AFR, where the reaction mixture flows through a series of heart-shaped cells to provide enhanced mixing designed to optimize and quickly scale up from lab-scale flow process development to large-scale industrial continuous production. On consideration of scale-up, the most efficient or sustainable process becomes important to consider. There is a choice of different processes, such as batch or continuous, and within these choices, a multitude of factors are to be considered. The purpose of this work was to take a relatively well-understood process that has been used commercially for over 30 years to evaluate how the different process conditions affected both the product properties, and to help guide the user in determining which process might be most suitable.

Cobalt-mediated catalytic chain transfer polymerization (CCTP) is a widely exploited free radical polymerization method and, unlike most controlled radical polymerization methods, has been used for over 30 years by a number of multinational companies, including ICI/DSM, DuPont, and 3 M, for a variety of commonly used products.^51–58^ It offers an easy and versatile pathway for the preparation of low molecular weight polymers with controlled molecular weight and a terminal vinyl group, which can be optionally used for post-functionalization or, sometimes, further polymerization. Due to its high efficiency, only parts-per-million levels of these low-spin cobalt­(II) catalysts are required to mediate an efficient process. According to the commonly accepted mechanism, CCTP is a two-step process: first, the Co­(II) complex abstracts a hydrogen from the growing radical, leading to a ω-vinyl-terminated product, often referred to as a *macromonomer*, and a Co­(III)–H complex; the Co­(III)–H complex reacts with a further monomer to regenerate the original Co­(II) complex and a propagating radical, enabling further chain growth.^59–66^ CCTP is effective for a range of vinyl monomers but is most effective with α-methyl-substituted monomers, such as methacrylates. The effectiveness of a chain transfer agent is measured by the chain transfer constant, *C*_s_, defined as the ratio of the rate constant for the chain transfer reaction to the rate constant for propagation (1):

1 $$C_{s}=k_{trs}/k_{p}$$

The chain transfer constant is determined using the Mayo equation; eq 2:

2 $$\frac{1}{DP_{n}}=\frac{1}{DP_{n0}}+C_{s}\frac{\left[S\right]}{\left[M\right]}$$

This present study focused on investigating CCTP reactions employing CoBF as the catalyst, performed in batch, a CSTR cascade, and tubular reactors under various conditions, and comparing reaction and product characteristics. Specifically, a commercial CSTR cascade system, referred to as the Scalable Agitated Baffle Reactor (SABRe) (Figure 1), a commercial Vapourtec tubular reactor, and a Corning Advanced Flow Reactor (AFR) were utilized. Corning’s AFR is a uniquely designed plate reactor with a series of heart-shaped mixing chambers (Figure 2), through which the reaction mixture is pumped. Each of these reactors was evaluated and compared to batch polymerization.

**Figure 1:**
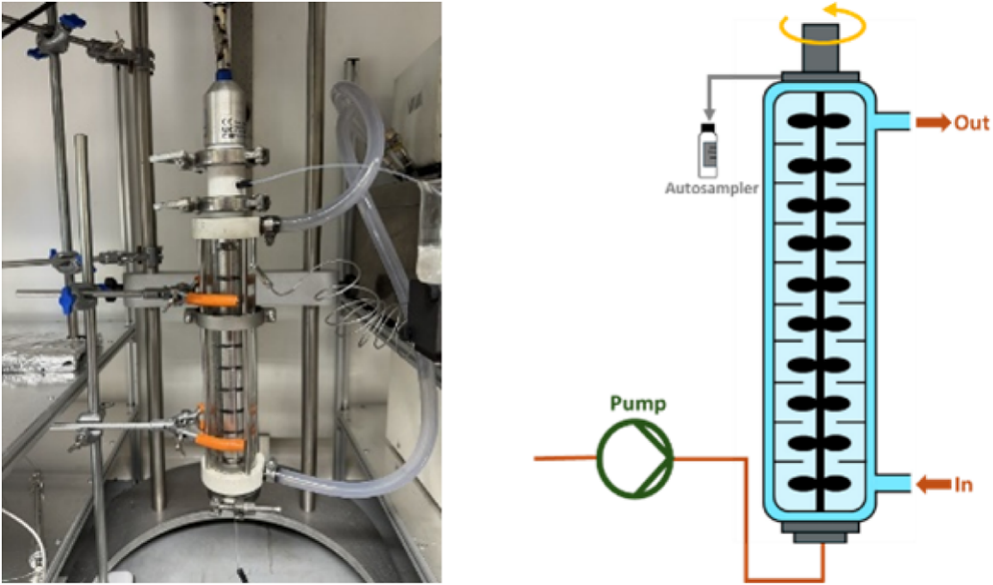
Schematic of the SABRe CSTR cascade reactor.

**Figure 2:**
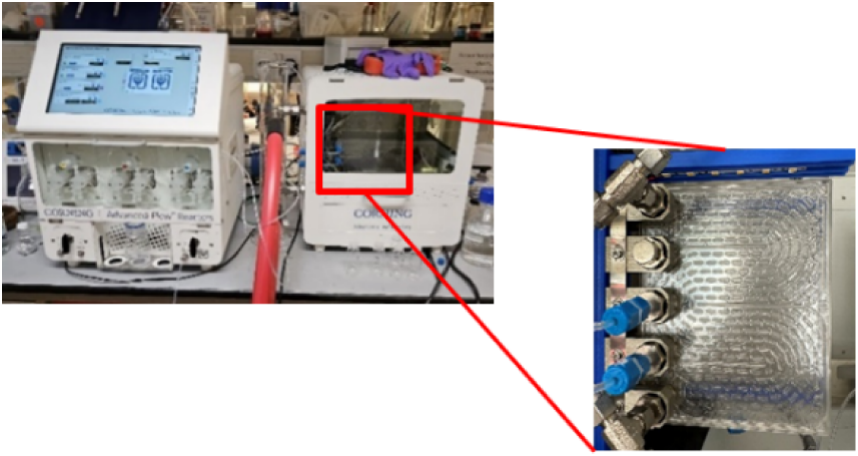
Corning advanced flow reactor.

The residence time distribution (RTD) was established for the systems to characterize the flow dynamics. Various monomers were utilized in the polymerization process, and reaction parameters were systematically varied to maximize reaction efficiency. The results were analyzed and compared to evaluate the feasibility and functionality of each flow system.

The purpose of this work was to compare a number of different flow reactors to see how they operate in an academic laboratory with a chemical process that is well established. As the chain transfer constant, as measured via the Mayo equation, is used, we naturally targeted low monomer conversion, as is required for this analysis, not seeking to provide a comparison for any industrial processes. Each reactor had different constraints in terms of flow rates/residence times, temperature limits, and control, which make exact comparisons difficult; however, we hope the work provides useful indications into a range of equipment available to study reactions in flow, with a view to both scaling up and for collecting real-time data for use in real-time process control.

## Experimental Section

### Materials

Materials and chemicals were purchased from commercial suppliers and used without additional purification unless otherwise stated. Methyl methacrylate (MMA, 99%, ≤30 ppm MEHQ as inhibitor), *n*-butyl methacrylate (*n*-BMA, 99%), benzyl methacrylate (BzMA, 96%), and glycidyl methacrylate (GMA, 97%) were purchased from Sigma-Aldrich and stored in a fridge at 4 °C. All monomers were purified by passing through a short column of basic alumina (VWR Chemicals) before use. 2,2′-Azobis­(2-methylpropionitrile) (AIBN, 98%) was purchased from Sigma-Aldrich. Toluene, tetrahydrofuran (THF), and deionized water were purchased from Sigma-Aldrich and Fisher Scientific.

### Characterization

Offline UV spectra were measured by injecting 200 μL samples into a 96-well, COC, F-bottom UV-STAR microplate and sampled in a BioTek Synergy HTX Multimode Reader, with inline measurements recorded on a Knauer WellChrom Spectro-Photometer K-2501 instrument fitted with a 10 μl (ID channel: 1:1 mm, 10 mm path length) stainless steel analytical flow cell (P/N: A4061) and a deuterium UV lamp operating in the wavelength range of 190–740 ± 2 nm. Device control and data communication were carried out via the RS-232 interface using custom-developed Labview drivers, with a data processing period of 80 ms. ^1^H NMR was conducted by dissolving samples in deuterated chloroform (CDCl_3_) (internal standard: 7.26 ppm) at 25 °C and recording on a Bruker Avance III HD 400 MHz NMR spectrometer. Chemical shifts are reported in ppm and data analyzed using ACD/NMR data software. The number-average molar mass (*M*_n, GPC_), weight-average molecular mass (*M*_w, GPC_), and the molecular weight distribution (dispersity) (MWD, *M*_w_/*M*_n_) were determined using an Agilent 1260 Infinity II-MDS equipped with differential refractive index, light scattering, UV–vis, and viscosity detectors, with two PLgel Mixed-D columns and a guard column. THF with a 0.01% w/v BHT (butylated hydroxytoluene) antioxidant was used as the eluent. Twelve PMMA narrow molecular weight standards were used for calibration, and all samples were prepared by diluting the polymer samples (0.3 mL) into THF (0.7 mL). Samples were filtered through a 0.2 μm PTFE syringe filter (Fisher).

### StoliChem 20 mL Scalable Agitated Baffle Reactor – CSTR Cascade Reaction System

The 20 mL Scalable Agitated Baffle Reactor (SABRe) was connected to a calibrated Huber Ministat 125 for cooling and heating circulators/baths for temperature control. A calibrated SIMDOS 10 FEM 1.10 S KNF liquid dosing diaphragm pump, equipped with a PVDF head, was utilized for reagent feeding, while an overhead stirrer was employed with temperature measured using a K-type probe inserted into the inner column.

### Corning Advanced-Flow Reactor (AFR)

The Corning AFR 2 instrument was equipped with three PTFE HPLC pumps for reagent feeding (Figure 2a). Reactions were conducted in two quartz 2.7 mL “sandwich structure” tightly integrated reactive layers with multiple heart-shaped mixing chambers. This reactor is designed to be operated from −40 to 200 °C and to hold up to 18 bar pressure. The temperature was controlled with a Huber Ministat 230 and a Minichiller 280 thermostat. This is a commercial system that has been designed to quickly and seamlessly scale up from lab-scale flow process development to large-scale industrial continuous chemical production. The flow reactor is in the form of a series of heart-shaped cells within a quartz plate fluidic module and operates at flow rates between 2 and 20 mL min^–1^ at temperatures between −40 and +200 °C, with an internal volume of 2.7 mL at up to 18 barg pressure.

### Vapourtec Flow System

A Vapourtec E-series flow chemistry system was equipped with two V-3 peristaltic pumps for reagent delivery, Figure 3 delivering a constant flow rate through a 10-mL stainless steel coiled tubular reactor.

**Figure 3:**
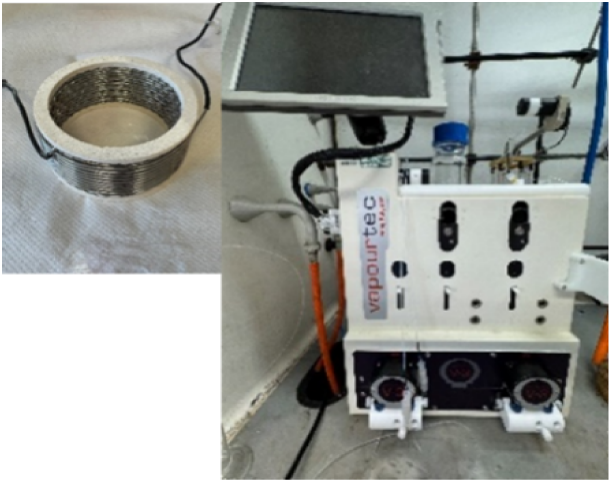
Vapourtec series E.

### Batch Polymerization of Alkyl Methacrylates (AMAs)

Batch polymerizations of methyl methacrylate (MMA), *n*-butyl methacrylate *(n*-BMA), and benzyl methacrylate (BzMA) were carried out under a nitrogen atmosphere. In a typical experiment, 9.6 mg of CoBF (2.5 × 10^–3^ mmol) was added to a 100-mL round-bottom flask and dissolved in 50 g (53.2 mL, 0.5 mol) of MMA. The mixture was sonicated and vortexed for 10 min to form a homogeneous orange CoBF stock solution. 125 mg (0.75 mmol) of AIBN was added to a second 100 mL flask, and all reagents were dissolved in 50 g (57.7 mL, 0.54 mol) of toluene. The contents were stirred until a homogeneous solution formed. Different amounts of stock solution were prepared in six separate 3-mL glass vials containing CoBF dissolved in MMA with a fixed amount of AIBN stock solution. The vials were vortexed by magnetic stirring bars for 10 min to form homogeneous solutions and then purged by bubbling nitrogen for 10 min. The sealed vials were placed in a preheated oil bath at 70 °C and left to react for 20 min while stirring at 200 rpm. Reactions were then quenched by placing the vials in an ice bath. Experiments were performed at higher temperatures of 80 and 90 °C to assess the temperature dependence, while maintaining all other experimental parameters constant.

For the reactions of BMA, 0.68 mg of CoBF (1.8 × 10^–3^ mmol) was dissolved in 50 g (56.0 mL, 0.35 mol) of BMA. BzMA polymerization was carried out with 50 g (48.1 mL, 0.28 mol) of BzMA and 0.54 mg (1.4 × 10^–3^ mmol) of CoBF, respectively, under the same conditions as for MMA.

### Continuous Flow CCTP in a Vapourtec Coiled Tubular Reactor, SABRe, and a Corning AFR

For the polymerization of MMA in the Vapourtec system (Figure 3), two stock solutions were prepared in two separate 250-mL round-bottom flasks. Stock solution A contained 100 g (106.4 mL, 1.0 mol) of MMA, 100 g (115.4 mL, 1.1 mol) of toluene, 250 mg (1.5 mmol) of AIBN, and 1.92 mg (5 × 10^–3^ mmol) of CoBF; stock solution B contained 100 g (106.4 mL, 1.0 mol) of MMA, 100 g (115.4 mL, 1.1 mol) of toluene, and 250 mg (1.5 mmol) of AIBN. The two stock solutions were sonicated and vortexed for 30 min to form homogeneous solutions, and then deoxygenated by bubbling with nitrogen for 30 min. To vary the amount of CoBF continuously, the ratio of the flow rates was controlled using two different peristaltic pumps for solutions A and B, while the total flow rate was kept constant (Table 1 and Figure 4). The reaction time (20 min) and temperature (70 °C) were the same as those for the batch reactions. Experiments were also carried out at 80 and 90 °C, with all other parameters held constant.

**Table 1:** Flow Reactions with Varying Flow Rates to Adjust CoBF Catalyst Concentration

CoBF (ppm)	Flow rate A (mL/min)	Flow rate B (mL/min)
0	0	1.25
1	0.25	1.00
2	0.50	0.75
3	0.75	0.50
4	1.00	0.25
5	1.25	0

**Figure 4:**
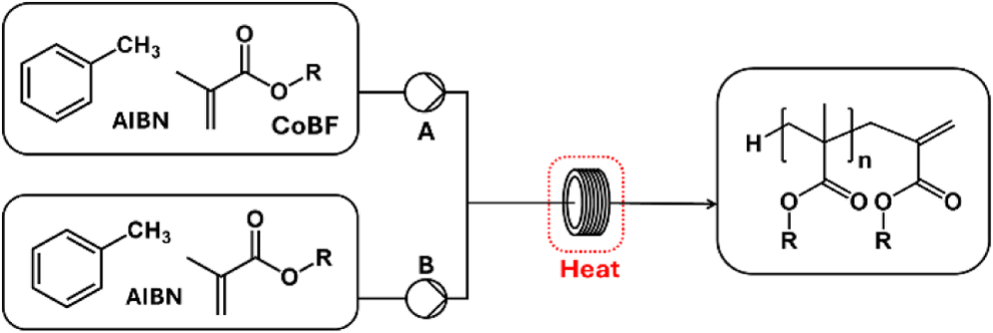
Schematic of the flow process using two peristaltic pumps (A and B) in an SS coiled reactor.

For the reaction of BMA, stock solution A was changed to contain 1.36 mg of CoBF (3.6 × 10^–3^ mmol) dissolved in 100 g (112.0 mL, 0.70 mol) of BMA. Separately, 100 g (96.2 mL, 0.56 mol) of BzMA with 1.08 mg (2.8 × 10^–3^ mmol) of CoBF was used as stock solution A in a further experiment. All other conditions were kept constant, as shown in Table 1.

For polymerizations carried out in the SABRe and Corning AFR reactors, 9.6 mg of CoBF (2.5 × 10^–3^ mmol) was added to a 100-mL round-bottom flask and dissolved in 50 g (53.2 mL, 0.5 mol) of MMA. The mixture was deoxygenated and vortexed for 30 min to form a homogeneous orange CoBF stock solution. The stock solution was kept under nitrogen prior to use. Toluene (200 mL) was added to a 250 mL flask and deoxygenated. AIBN (300 mg, 1.8 mmol) was added to a second 250 mL flask, and all reagents were dissolved in 120 g (138.4 mL, 1.3 mol) of toluene. The contents were stirred until a homogeneous solution formed. Different stock solutions were prepared in six separate 50-mL round-bottom flasks containing CoBF dissolved in MMA, with a fixed amount of AIBN stock solution added. The reagents were vortexed using magnetic stirring bars to form homogeneous solutions and purged by bubbling nitrogen for 20 min. For reactions in the SABRe, nitrogen was purged through the reactor column before the reaction. The deoxygenated toluene was pumped using a KNF pump under nitrogen at 8 mL/min, which was quickly connected to the inlet port of the reactor for flushing through. The flushing process was conducted at a high stirring rate (400 rpm) to remove all nitrogen bubbles. The flow rate was decreased to 1 mL/min, and the flushing solvent was then switched to the deoxygenated reaction solution. The stirring rate of the SABRe system was set to 100, 200, and 300 rpm. The reactors were flushed with toluene following each reaction. For the Corning AFR, both reactor plates were flushed with deoxygenated toluene prior to the reaction. The temperature (70 °C) was maintained the same as for the batch reactions, while the residence time was set to 5 min. Experiments were also carried out at 80 and 90 °C, with all other parameters held constant.

### Online Monitoring of Continuous Flow Polymerization Using a Vapourtec E-Series Reactor System

A round-bottom flask was prepared with 100 g (93.5 mL, 0.70 mol) of GMA, 100 g (115.34 mL, 1.09 mol) of toluene, 250 mg (1.52 mmol) of AIBN, and 2.165 mg (5.6 × 10^–3^ mmol) of CoBF. This flask was deoxygenated with a steady stream of nitrogen bubbling for 30 min. The flask was then connected to the Vapourtec E-series device using 0.8 mm ID PFA tubing. A single peristaltic pump was used to deliver the reaction mixture at 0.5 mL/min through a 10-mL stainless steel tubular reactor suspended in an oil bath at 70 °C, for a total runtime of 3 h.

20 μL of this reagent stream was sampled every 15 min using a modified Agilent Infinity II instrument equipped with a differential refractive index (DRI) detector, a PLgel Mixed D column (300 × 7.5 mm^2^), and a PLgel 5 μm guard column with THF as the eluent. Samples were run at a flow rate of 1 mL/min at 30 °C. Poly­(methyl methacrylate) standards (Agilent EasiVials) were used for calibration. Experimental molar mass (*M*_n_, *M*_w_, and SEC) and dispersity (*Đ*) values of the synthesized polymers were determined by conventional calibration using Agilent OpenLabs software.

### RTD Using UV–Vis and RI Detection

Measurement of the residence time distribution (RTD) of the AFR and Vapourtec reactors was conducted using a rhodamine B solution in water (*c* = 1 × mol/L), with the peak absorption at λ = 554 nm as the reference. The UV spectra for the coiled tube reactor were recorded using inline UV measurement, while the spectra for the AFR were obtained using an offline UV machine with an autosampler. The RTD of the SABRe was measured using a refractive index (RI) detector to monitor the THF signal in water, as the higher sensitivity of UV light led to unacceptable baseline fluctuations.

### RTD Test for the Corning AFR

A similar method was used with 20 μL of rhodamine B injected, considering the smaller volume of the reactor in the AFR, with a flow rate of 1.08 mL/min. The absorption peak associated with rhodamine B was observed at *t* = 8 min. The stability test of the AFR was conducted for 5 min, both with and without the catalyst, at 90 °C

### RTD Test for the Vapourtec System

The RTD for the Vapourtec was conducted by injecting 100 μL of rhodamine B and flushing water into an 18-mL coil tubular reactor (as measured), followed by UV detection with a flow rate of 1.25 mL/min. The absorption peak associated with rhodamine B was observed at *t* = 14.6 min. The stability test of the system was conducted by polymerizations for 20 min, with and without the catalyst, at 70 °C.

### RTD Test for the SABRe System

A 100 μL of THF in water solution was injected into the system at a pumping speed of 1 mL/min and a stirring rate of 100 rpm. Samples were collected at the outlet of the SABRe tube, and the RI signal of THF was measured. The stability test of the system was conducted by polymerizations for 20 min with and without the catalyst, at 70 °C under 100 rpm.

## Results and Discussion

### Residence Time Distribution Test for Flow Systems

The residence time distribution (RTD) describes the time profile that a fluid or solid particle spends passing through a reactor in a given experimental set up (Figure 5). This provides insights into the characteristics of the flow regime, mixing behavior, flow delays, and other related effects. RTDs are reactor- and process-specific, varying with reaction setup, solvent choice, temperature, and viscosity, and as such, are always an important parameter to measure and consider when carrying out experiments and comparing reactors.

**Figure 5:**
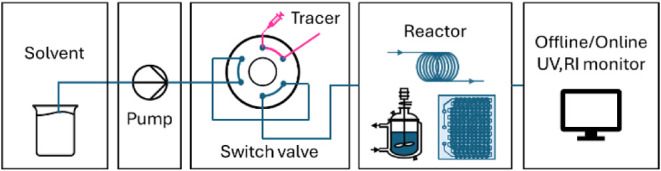
RTD tests with the tracer injection method based on offline and online UV and RI detection.

Both offline (Figure 6) and inline (Figure 7) UV spectroscopy approaches were employed for obtaining the RTD of Corning’s AFR and Vapourtec PFR, respectively.

**Figure 6:**
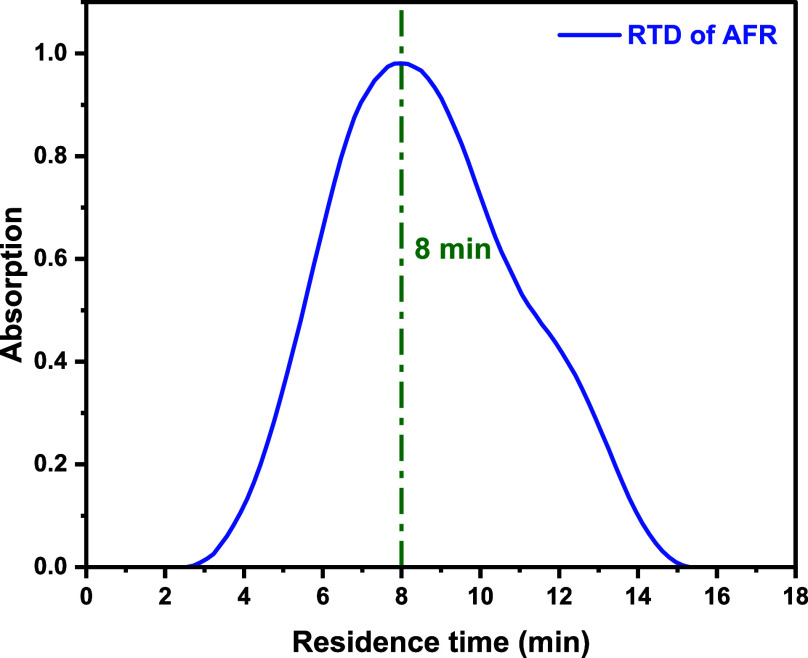
Determination of RTD using tracer (rhodamine B) analysis via UV–vis response for Corning’s AFR.

**Figure 7:**
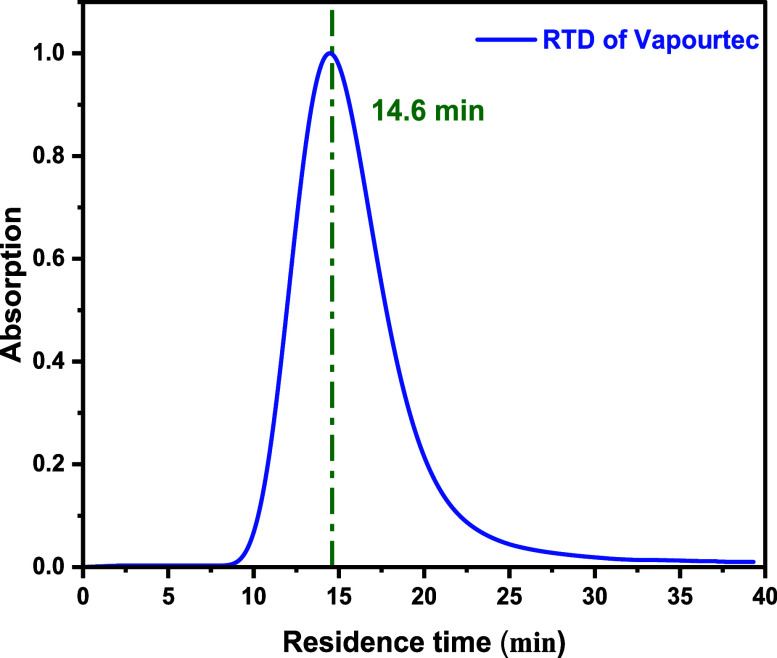
Experimental determination of RTD using rhodamine B UV–vis for the Vapourtec PFR.

A refractive index detector (RID) was also used as an alternative approach in an online configuration for RTD of the SABRe system, due to baseline fluctuations in UV with higher sensitivity (Figure 8).

**Figure 8:**
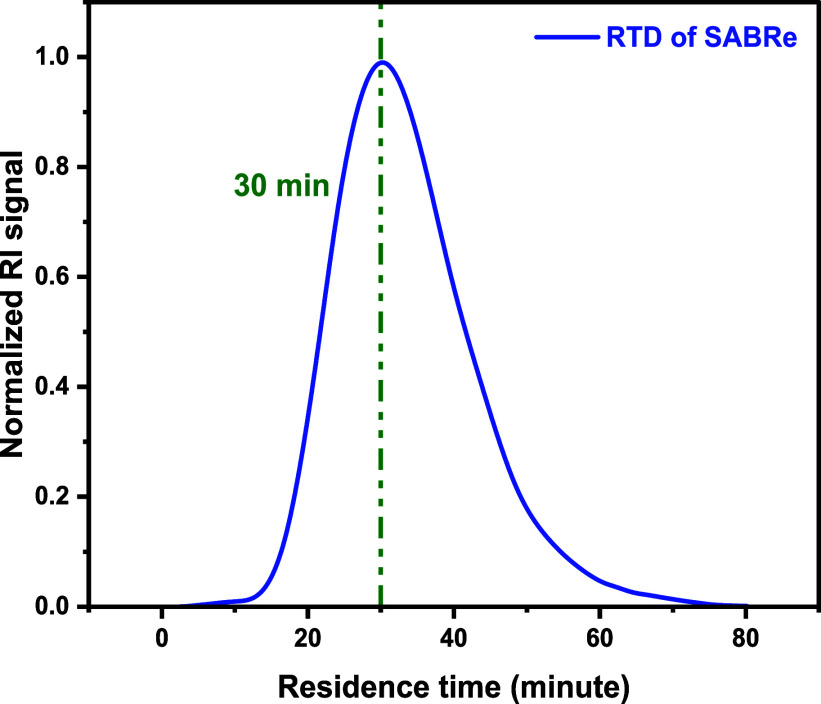
Experimental determination of the RTD using THF analysis and an RI response for the SABRe system.

### Interpreting RTDs

Both the average residence (τ_*r*_ and Peclet number (Pe) can be determined from these RTD traces. The Peclet number is an important dimensionless unit that describes the mixing of a fluid traveling through a volume. In short, the axial transport rate is divided by the longitudinal transport rate. This means that it can be used as an approximation for the mixing within a flow reactor. Low values of Peclet (Pe < 1) are indicative of a flow regime where diffusion dominates the mass transfer. When Pe = 1, axial and longitudinal transport equally contribute to mass transfer, and as Pe tends to ∞, longitudinal transport dominates. Pe values above 100 are viewed as being in a quasi-plug flow.

The axial dispersion model was used for fitting the *E*(*t*) based on eq 3:^67^

3 $$E\left(\theta \right)=\sqrt{\frac{\mathrm{Pe}}{4\pi \theta }}e^{[-{\left(1-\theta \right)}^{2}\mathrm{Pe}/4\theta ]}$$

Where Pe = Peclet number, $\theta =\frac{t}{\tau_{r}}$ , and τ_*r*_ is the average residence time of the reactor

A nonlinear least-squares analysis was performed to fit eq 3 to the experimentally determined RTD in order to obtain the parameters Pe and τ_r_ (Table 2). Exemplars of these fits can be found in the Supporting Information. The determination of the Peclet number and the use of the fitting algorithm will be discussed further in an upcoming publication from our working group.

**Table 2:** Determination of Average Residence Time and the Peclet Number

System	Flow rate (mL min^–1^)	Volume (mL)	τ_*r*_	Pe
SABRe	1	20	31.2	25.24
AFR	1.08	5.4	8.2	21.75
Vapourtec PFR	1.25	18	14.6	66.44

The calculated results of each system’s parameters are shown below:

The disparity between the expected average residence time and experimentally determined times highlights the need for experimental determination of these values. In the cases of the Corning AFR and the SABRe reactors, the broader RTD curves indicate that there are more dead volumes within the reaction line compared with the Vapourtec PFR (Figures 6–8). The greater variance in reagent concentration, as a result of larger dead volume, may lead to a higher dispersity of polymer products. As expected, the calculated Peclet numbers for these two reactors were lower than those of the coil tubular reactor, indicating less ideal plug-flow-like character. These values indicate that the Corning AFR behaves more similarly to a CSTR cascade than an ideal plug flow reactor.

### Polymerization of Methacrylates in Different Flow Reactors

Polymerizations of methyl methacrylate (MMA) in toluene were conducted following a standard procedure using all three flow platforms to directly compare how the different flow regimes affect the product of that reaction. Initially, free radical polymerization was employed using AIBN as the initiator. Reagents were delivered to the reactors with a residence time of 20 min in the SABRe and coil reactor at 70 °C, and 5 min in the AFR at 90 °C. The reaction flow was then held at a steady state for 30 min before switching to a continuous solvent flow. Samples were collected from the reactor output every 5 min following the onset of the steady-state condition. This was carried out by manually diverting the output flow to a collection vessel and collecting a sample for 30 s. Conventional offline GPC analysis was performed by using THF as an eluent. Each system produced polymers with a relatively consistent product, as measured by mass, with a standard deviation of 1.6 K g mol^–1^ for the Vapourtec, 1.0 K g mol^–1^ for the Corning AFR, and 1.1 K g mol^–1^ for the SABRe, each with a coefficient of variance of less than 4% (Figure 9). Full GPC spectra can be found in the Supporting Information. These results show that each system produced polymers with very similar molecular weight characteristics and excellent reproducibility. This is as expected, and once the system reached a steady state, the chemistry should proceed without variance.

**Figure 9:**
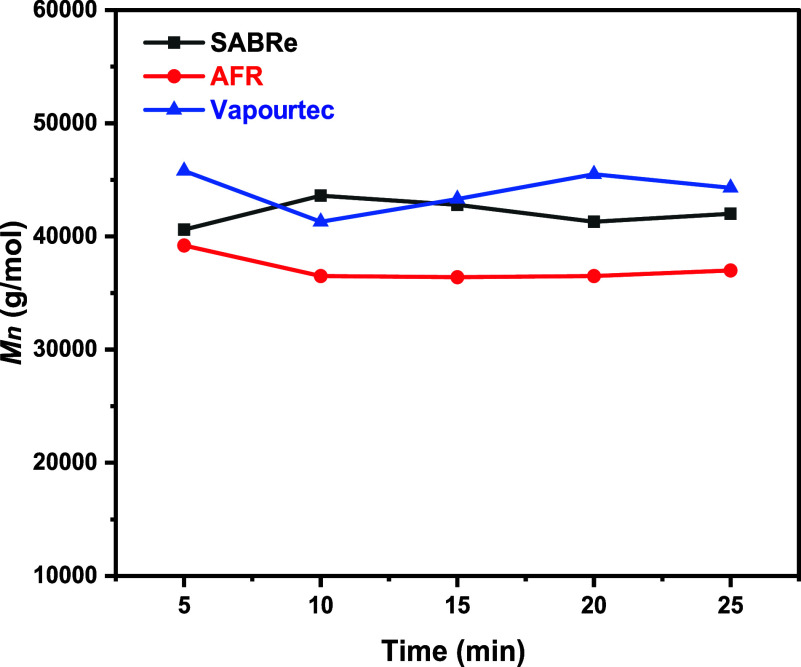
GPC data for the free radical polymerization of MMA in toluene were obtained in different reactors. Samples were collected every 5 min after the average residence time.

Following on from this, CCTP of MMA in toluene was conducted under similar reaction conditions with the addition of 1 ppm of CoBF as a chain transfer agent (CTA). Similar results were found in Vapourtec PFR and SABRe. A small variation in molecular weight for the CCTP with CoBF in the AFR was observed, which might be attributed to diffusion within the reactor plate and between the outlet collector and reactor at low flow rate (Section S2.1.1)

The dispersity of polymers collected from the SABRe and coil reactor was compared to gain insight into the RTD, as shown in Figure 10. It was observed that the dispersity of polymers produced in the SABRe reactor was not consistently higher than that from the plug flow reactor (PFR), despite its broader RTD. This outcome may be attributed to more efficient mixing within the SABRe system, as indicated by its lower Peclet number. Additionally, at low monomer conversions, the monomer concentration remains nearly uniform, and radical concentrations are relatively low, which likely minimizes the effect of spatial variations on molecular weight distribution during free radical polymerization. Consequently, no definitive correlation between RTD and polymer dispersity was established. This is further supported by experimental data (Supporting Information), where increasing the stirring rate in the CSTR did not lead to a reduction in dispersity.

**Figure 10:**
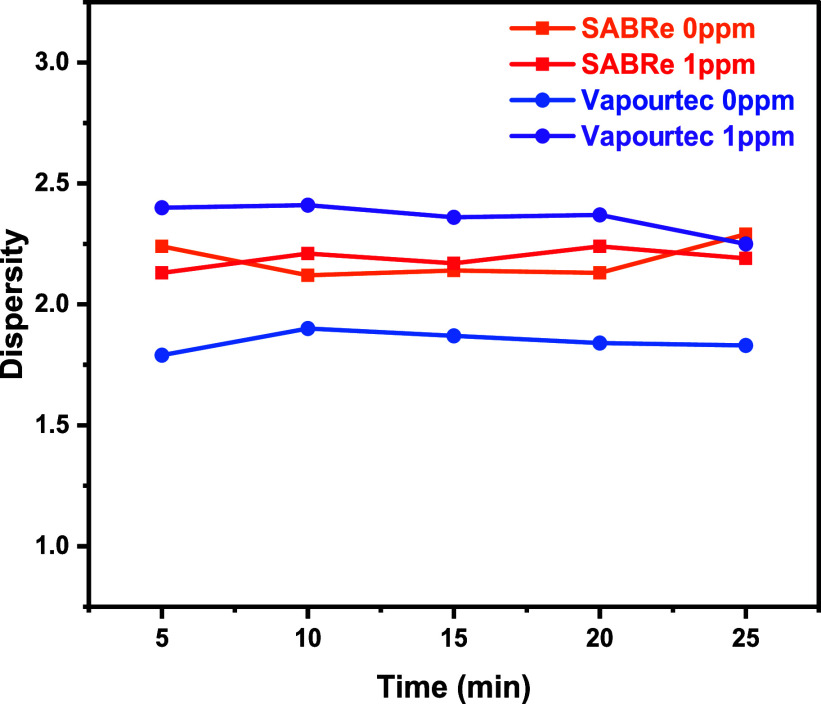
Polymer dispersity changes before and after the addition of CoBF catalyst in the Sabre and Vapourtec systems (SF2 and SF3).

The reproducibility of the experiments was further verified by the continuous polymerization of GMA with 8 ppm of CoBF at 70 °C, monitored by online GPC (Figure 11). This was accomplished by flowing the output of the flow reactor into a GPC inlet valve. 20 μL of the reaction mixture was injected every 15 min into an Agilent mixed D GPC column set, and data were collected using an RID over a 180-min period. Further details on how to accomplish this will be the subject of a series of detailed future publications. We note that there was no dilution of the reaction mixture prior to column injection.

**Figure 11:**
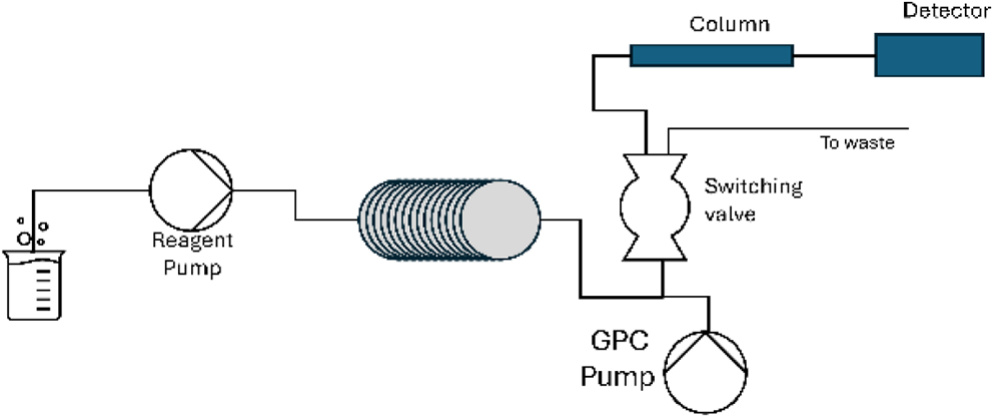
Configuration of the online GPC for reaction monitoring of the output of a flow reactor.

Throughout the 3-h reaction, the flow reactor produced identical products (Figure 12 with polymer peaks between 8 and 10 min retention time), demonstrating a robust process with high reproducibility.

**Figure 12:**
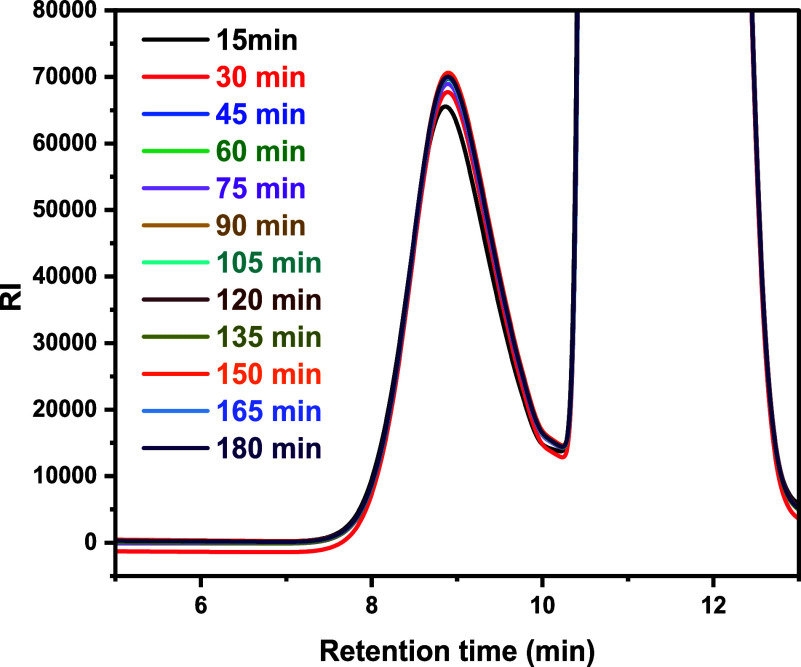
GPC traces (12) of samples from CCTP of GMA with 8 ppm of CoBF under 70 °C, collected every 15 min for 3 h. *M*_w_ ∼3000 g/mol. The large signals after 10 min were due to residual toluene and monomer in the reaction.

### Effect of Stirring Rate

Mixing in this type of reaction can be important, especially since the viscosity of the reaction medium increases throughout the reaction as the concentration of polymer increases. The mixing effect of the agitators in SABRe was investigated. As the stirring rate increases, the flow pattern in stirred tanks becomes turbulent, as predicted by the dimensionless impeller Reynolds number.^68^ Thus, the stirring speed in the CCTP polymerization process using the bench-scale SABRe module was carried out at 100, 200, and 300 rpm (Figure 13 and Table 3).

**Figure 13:**
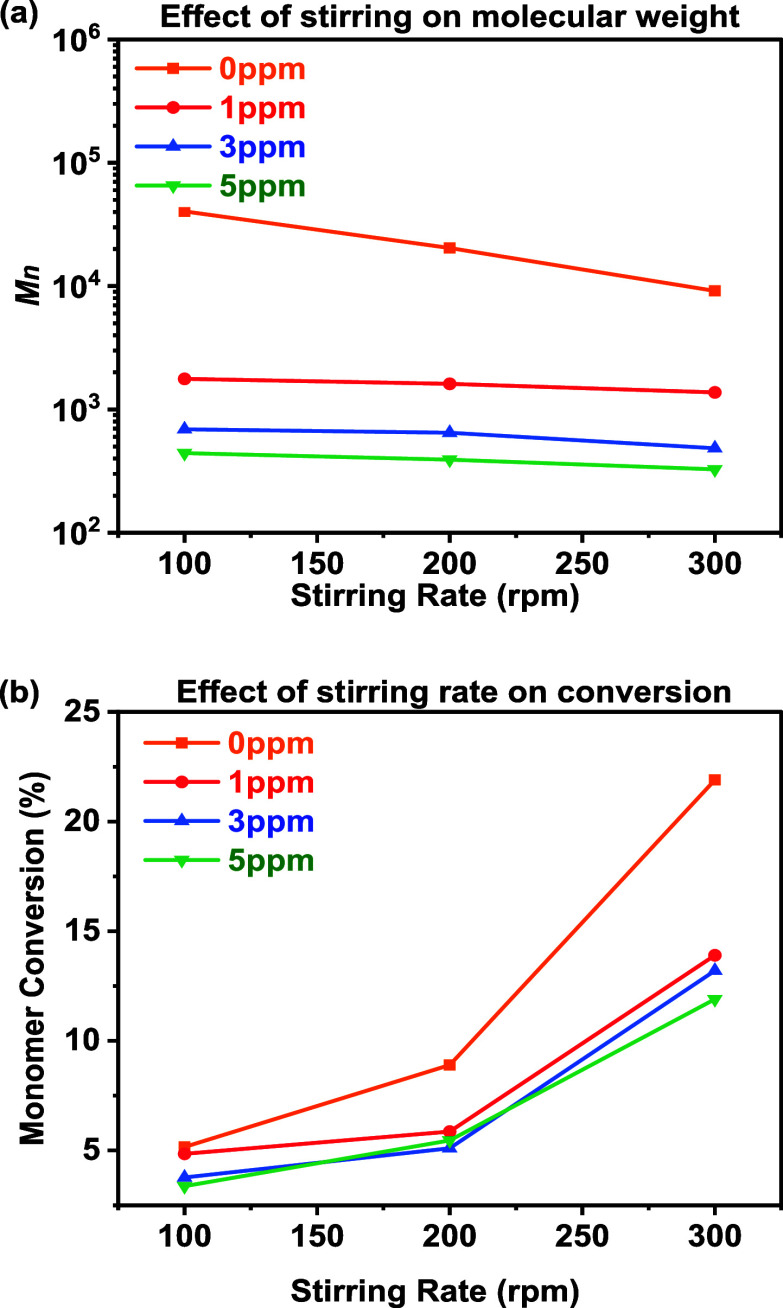
(a) log (*M*_*n*_) change of samples and (b) monomer conversion change of samples under 100, 200, and 300 rpm stirring rates, collected in the presence of different [CoBF].

**Table 3:** Effect of Stirring Rate on the Chain Transfer Constant of CoBF with MMA at 70 °C

Stirring rate (rpm)	Conv. (%)	*C*_s_
100	3.4–5.2	42200
200	4.6–8.9	46800
300	11.9–21.9	49600

As the stirring rate increased, the monomer conversion also increased, while the molecular weight decreased. In the CSTR cascade, the reactants are constantly diluted with the product under constant stirring, so the reagent concentration remains constant. The enhanced mixing efficiency increased the collision frequency between growing radicals and monomers, resulting in the increase of conversion and lower chain length, which shall be differentiated from the PFR, where the ratio of radical concentration to monomer concentration increases gradually along the reactor length, yielding similar product characteristics despite differing flow dynamics. The *C*_s_ value remained quite constant with faster stirring, as better mixing ensures a more uniform distribution of the cobalt­(II) complex, enabling more consistent reversible deactivation of radicals. Theoretically, increasing the stirring rate improves mixing, reduces the impact of dead zones or bypassing, and leads to a narrower RTD, potentially resulting in lower dispersity.^50^ However, no clear correlation between polymer dispersity and stirring rate was observed, as stated before (see the Supporting Information).

### Determination of the Chain Transfer Constants for the CCTP of MMA

CCTP was conducted in both batch and with three different flow reactors to examine the influence of operational conditions on the synthesis of polymethacrylates. Specifically, variations in temperature, monomer type, and stirring rate in the different flow reactors, and their effects on monomer conversion and the efficiency of chain transfer were examined. The effect of temperature on the CCTP process was studied in the SABRe, Vapourtec, AFR, and in batch between 70 and 90 °C. As in conventional radical polymerization, the molecular weight should decrease with (a) an increase in the amount of chain transfer agent relative to monomer and (b) an increase in the [radicals], which can be brought about by either increasing the [initiator] or raising the temperature, as the rate of termination is second order in [radicals] and chain growth is first order.

### Batch Polymerization

Batch polymerizations of methyl methacrylate (MMA) were carried out in 3 mL vials, with the small volume reducing the effect of slow heat conduction, which is possible in bulk reactions relative to tubular flow reactors (Table 4). Compared with flow reactions in the SABRe, AFR, and Vapourtec systems at 70 and 80 °C, the *C*_s_ values in batch reactions are higher at similar monomer conversions. The *M*_w_/2 was used as a measure of the molecular weight rather than *M*_n_ to reduce the large uncertainty caused by baseline errors in the GPC, causing *M*_n_ to deviate more than *M*_w_/2, as suggested by Davis.^69^

**Table 4:** Chain Transfer Data for the CCTP of MMA in Toluene Conducted at 70, 80, and 90 °C, in Batch and the Three Commercial Flow Platforms

Reactor	Temp (°C)	Conv. (%)	*C*_s_
Batch	70	3.2–4.7	58700
Batch	80	6.7–11.7	47400
Batch	90	13.3–26.1	35300
SABRe	70	3.4–4.9	42200
SABRe	80	7.0–10.7	48400
AFR	70	3.4–5.0	21600
AFR	80	4.8–6.0	24800
AFR	90	4.7–6.6	24100
Vapourtec	70	2.9–6.0	47600
Vapourtec	80	11.0–13.0	42000
Vapourtec	90	19.4–23.5	42800

At a fixed [MMA]/[CoBF], the monomer conversion increased, and the *M*_w_ decreased, as would be expected for a free radical polymerization, with the measured *C*_s_ value decreasing at higher temperatures. A direct comparison is difficult as the Mayo equation assumes that the [MMA]/[CoBF] ratio is constant; however, as conversion increases, [MMA] decreases while [CoBF] remains relatively constant, causing this ratio to vary since [CoBF] is not consumed in the reaction. The decrease in measured chain transfer constants with increasing temperature is somewhat surprising, given that this has been previously shown to be temperature-independent. We note that we also see an increase in conversion to over 10%, whereas the Mayo equation assumes close to zero monomer conversion. Additionally, at higher temperatures, we might have seen increased catalyst decomposition under the reaction conditions used.^70–72^ The presence of CoBF should not reduce the rate of polymerization, which would lead to lower monomer conversions, which is not expected for chain transfer in FRP but is in agreement with previous work and aligns with the observed reduction in molecular weight.^59,60^

### SABRe-CSTR Cascade

The influence of temperature was also tested for the SABRe system at 70 and 80 °C at 100 rpm, as shown in Table 4. Here, we have vastly increased mixing through the reactor with 10 independent mechanical stirrers within the flow tube. For normal CSTR cascades, when reagents are fed into the chamber, the monomer concentration is quickly diluted, which can lead to lower local radical concentration and propagation. However, for the SABRe system, the small volume of each mixing chamber reduced the dilution impact and ensured a decent monomer conversion. The molecular weight for the system was smaller than that for the tube reactor due to the increased likelihood of chain transfer or termination under more efficient mixing. (Supporting Information) The *C*_*s*_ values remained quite similar to an increase in temperature, similar to the Vapourtec system with the simple tubular reactor. No effect of the temperature increase on product dispersity was observed. Volatilization of reagents at 90 °C resulted in the formation of a substantial number of bubbles within the reactor, thereby making the process of sample collection difficult.

### AFR

The effect of temperature in the AFR was tested at 70, 80, and 90 °C. This reactor has been designed to provide extremely efficient mixing at its core design principle but is only suitable for relatively short residence times, making direct comparisons not possible. Therefore, the temperature was increased to obtain relative data at sufficiently high conversions. A similar trend was observed; however, it is noted that the chain transfer constant was lower than those obtained from the other two systems. Although we are not sure of the cause, it could be due to a small amount of air/oxygen being trapped in this case, which is known to lead to degradation of the CTA and lower measured *C*_s_ values, or due to mixing effects (Figure 14). It was somehow verified when the reagents were introduced at a lower speed (0.27 mL/min), resulting in poor control over the polymer’s molecular weight. The *C*_s_ value was consistent across all the temperatures studied, as the residence time was kept constant.

**Figure 14:**
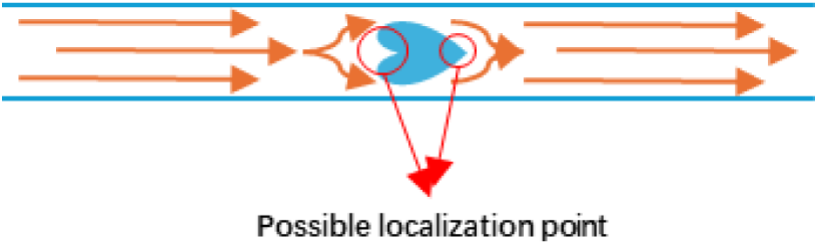
Diagram of the possible localization of CTA in the reactor at a low flow rate.

### Vapourtec Flow System

The effect of changing the temperature in the Vapourtec flow system was also studied. In these experiments, temperature had no significant influence on the chain transfer efficiency. It is noted that conversions were also increasing, but the *C*_s_ values calculated based on Mayo plots for 70 , 80, and–90 °C were found to be 47600, 42000, and 42800, respectively. The monomer conversions increased, and the molecular weight of the obtained polymers decreased at higher temperatures, as expected. On this basis, we obtained very comparable results to batch polymerization when carrying out flow reactions in a simple tubular reactor in which the reagents were driven by laminar flow. The lack of mixing has little effect on the dispersity of the product, as discussed, within a short residence time, and monomer conversion remains quite similar.

### Different Types of Methacrylates

The effect of changing the methacrylate was investigated, and it is noted that previous work has shown that *C*_s_ decreases with an increase in steric hindrance and possible changes in viscosity, with the order being MMA > BMA > BzMA.^70,72^

Batch polymerizations of butyl methacrylate (BMA) and benzyl methacrylate (BzMA) were carried out at 70 °C, as shown in Table 5. The calculated *C*_s_ values were 58700, 53800, and 22100, respectively. The feasibility of the SABRe system was studied by polymerizing MMA, *n*-BMA, and BzMA using the same residence time (20 min). The *C*_s_ values for the monomers follow the order: MMA > *n*-BMA > BzMA, which is similar to the observations in the batch and the Vapourtec flow system. CCTP for MMA, BMA, and BzMA was carried out at the same temperatures and flow rate to test the feasibility of the Corning AFR. A similar trend was observed with MMA, again showing somewhat reduced chain transfer constants, which may be due to either a higher amount of oxygen/air in the reaction resulting in some catalyst decomposition or catalyst localization, as discussed before. The reactions with MMA, BMA, and BzMA were carried out at the same temperature and flow rate using a peristaltic pump for additional reagents and to provide the flow. The *C*_s_ values showed the catalytic chain transfer activity of the different monomers, with *C*_s_ = 47600 for MMA, 28800 for BMA, and 19500 for BzMA. The trend of *C*_*s*_ values decreased as steric hindrance increased, as also observed with the SABRe system. Among the various reaction systems studied, the *C*_s_ values for all three monomers were highest in batch. This was attributed to the relatively efficient heat and mass transfer within the small vials immersed in the oil tank under vigorous stirring, and the differences observed were consistent with those already reported.^70,72^

**Table 5:** Chain Transfer Data for the CCTP of MMA, BMA, and BzMA in Toluene Conducted at 70 °C in Batch and the Three Commercial Flow Platforms

Reactor	Monomer	Conv. (%)	*C*_s_
Batch	MMA	3.2–4.7	58700
Batch	BMA	2.9–5.2	53800
Batch	BzMA	5.8–13.6	22100
SABRe	MMA	3.4–4.9	42200
SABRe	BMA	3.3–6.1	30600
SABRe	BzMA	3.1–5.3	27000
AFR	MMA	3.4–5.0	21600
AFR	BMA	4.7–5.9	18700
AFR	BzMA	4.1–5.4	16300
Vapourtec	MMA	2.9–6.0	47600
Vapourtec	BMA	3.7–6.5	28800
Vapourtec	BzMA	5.9–9.5	19500

### CCTP of GMA

To further validate the adaptability of the reaction system to a broader spectrum of monomers, and in particular reactive monomers, the Vapourtec system was employed for the polymerization of GMA. The [CoBF] was varied from 2 to 10 ppm with the reaction temperature set to 70 °C (Figure 15 and Table 6).

**Figure 15:**
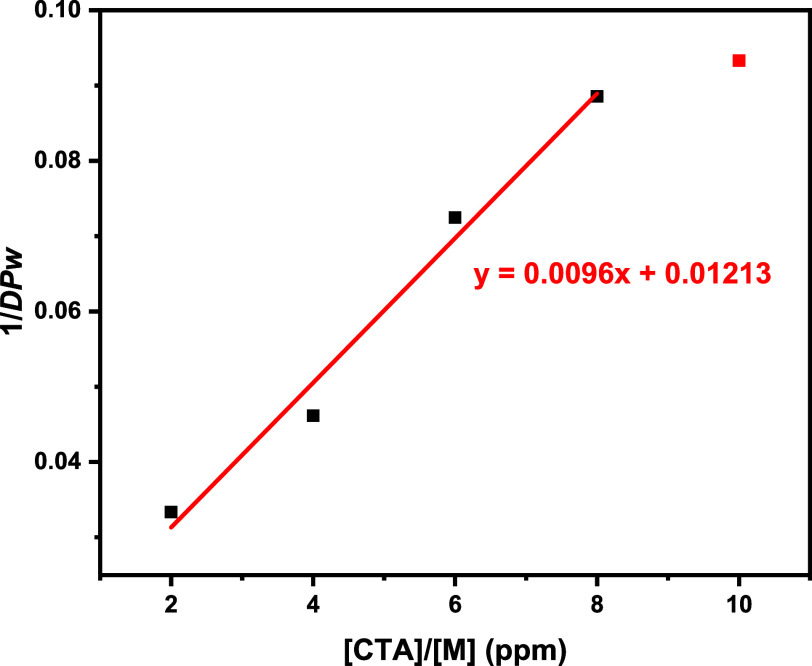
Mayo plot derived from GPC of collected samples from the CCTP of GMA with 2–10 ppm of CoBF.

**Table 6:** Weight-Average Molecular Weight (*M*_w_), Dispersity, Conversion, and *C*_s_ Value for Polymerization of GMA at 70 °C Using the Vaportec Reactor

Monomer	CoBF (ppm)	*M*_w_ (g/mol)	*Đ*	Conv. (%)	*C*_s_
GMA	2	8500	1.9	9.5	9600
	4	6200	1.9	8.8	
	6	3900	1.8	7.9	
	8	3200	1.8	7.5	
	10	3000	1.8	5.4	

The CCTP of GMA, in the absence of a catalyst, was not carried out in the flow system due to the potential for blockage/cross-linking at higher molecular weights. A slightly lower *C*_*s*_ value was observed, which could be attributed to the larger steric requirements and the potential coordination of the epoxy group with the cobalt active site, which might decrease catalyst efficiency.

## Conclusions

We report the use of three different flow processes and compare them with batch polymerization, all of which give quite similar results. This demonstrates the effectiveness of the air-sensitive cobalt-mediated catalytic chain transfer polymerization (CCTP) being carried out under flow conditions using three distinct reactor designs: SABRe (CSTR cascade), a tubular flow reactor, and a Corning Advanced Flow Reactor (AFR). Each reactor showed different flow dynamics and mixing efficiencies, which somewhat impacted polymer characteristics such as molecular weight, dispersity, and monomer conversion rates, ultimately with an effect on the properties of the final products. These results highlight that different flow systems can achieve comparable control to analogous small-scale batch polymerization while also enabling continuous production, giving the advantage of carrying out the polymerization in a continuous fashion.

Temperature, monomer type, and stirring rate were key variables influencing reaction outcomes and final product properties. Higher temperatures and stirring rates generally increased monomer conversion while reducing the molecular weight.

Overall, the versatility of CCTP under flow conditions and the use of parts-per-million-level cobalt catalysts affirm the process’s potential for efficient, large-scale production of polymethacrylates with tailored properties. In addition, we show how online monitoring can be used to collect almost real-time molecular weight and molecular weight distribution data. The findings may support broader applications of flow polymerization in industrial settings.

## Supplementary Material



## Notes

Python script for curve fitting, fitted RTD curves, GPC traces of products, and tabulated molecular weight and conversion data (PDF)
