# Supplementary material for: Co(II)-Mediated Catalytic Chain Transfer Polymerization (CCTP) Carried Out Under Flow Reaction Conditions and Introducing a New Method for Online GPC Monitoring
Source: ACS Polym Au. 2025 May 29;5(3):311–22. doi: 10.1021/acspolymersau.5c00020 (PMC12163948; doi:10.1021/acspolymersau.5c00020)
Supplement: Supplementary file 1 [file lg5c00020_si_001.pdf]

Supporting information

Co(II) mediated catalytic chain transfer polymerization (CCTP) carried out under flow reaction conditions and introducing a new method for online GPC monitoring

Yanpu Yao*[a], Xiaofan Yang* [a], Cansu Aydogan [a], James Town [b], William Pointer [a] and David M. Haddleton [a]**

***These authors contributed to the work equally.**

[a] Department of Chemistry, University of Warwick, Coventry, CV4 7AL, United Kingdom

[b] Polymer Characterization Research Technology Platform, University of Warwick, Coventry, CV4 7AL, United Kingdom

****d.m.haddleton@warwick.ac.uk**

1. Python script and Fitted curve for each system:

1.1 Script

```
def run_calc():#

   RTD_data = pd.read_csv(r'YOUR DATA LOCATION', skiprows=1,
header=None)
   x_vals = RTD_data.iloc[:,0].values
   y_vals = RTD_data.iloc[:,1].values
   y_vals = (y_vals - np.min(y_vals)) / (np.max(y_vals) - np.min(y_vals))

   optimised_perams = RTD_CALC(x_vals,y_vals)
   plot_data(x_vals, y_vals, optimised_perams)


   print ("optimised_perams")


def RTD_CALC(x_vals,y_vals):

   def peclet_calc(params, x_vals, y_vals):
      Tr, Pe = params
      calc_y = (np.sqrt(Pe / (4 * np.pi * (x_vals / Tr)))) * np.exp(-(((1 - (x_vals/
Tr))**2) / ((4 * (x_vals / Tr)) / Pe)))
      norm_calc_y_vals = (calc_y - np.min(calc_y)) / (np.max(calc_y) -
np.min(calc_y))
      dif = y_vals - norm_calc_y_vals
      return dif

   init_t = get_x_of_max_y(x_vals,y_vals)
   print (init_t)
   initial_guess = [init_t, 4543]
   result = least_squares(peclet_calc, initial_guess, args=(x_vals, y_vals))

   optimized_params = result.x
   print("Optimized parameters:", optimized_params)
   return optimized_params

def get_x_of_max_y(x_vals,y_vals):
   idx_max_y = np.argmax(y_vals)
   return x_vals[idx_max_y]


def plot_data(x_vals, y_vals, optimized_params):
   plt.plot(x_vals, y_vals, label='RTD Data')
```

```
    # Calculate the fitted line using the optimized parameters
    Tr, Pe = optimized_params
    print (Tr)
    print (Pe)
    fitted_y_vals = (np.sqrt(Pe / (4 * np.pi * (x_vals / Tr)))) * np.exp(-(((1 - (x_vals/Tr))**2) / ((4 * (x_vals / Tr)) / Pe)))
    fitted_y_vals = (fitted_y_vals - np.min(fitted_y_vals)) / (np.max(fitted_y_vals) - np.min(fitted_y_vals))

    # Plot the fitted line
    plt.plot(x_vals, fitted_y_vals, label='Fitted Line', linestyle='--')

    plt.xlabel('time')
    plt.ylabel('detector response')
    plt.title('RTD trace')
    plt.legend()
    plt.show()
```

1.2 Fitted Curves

Fitted RTD of AFR

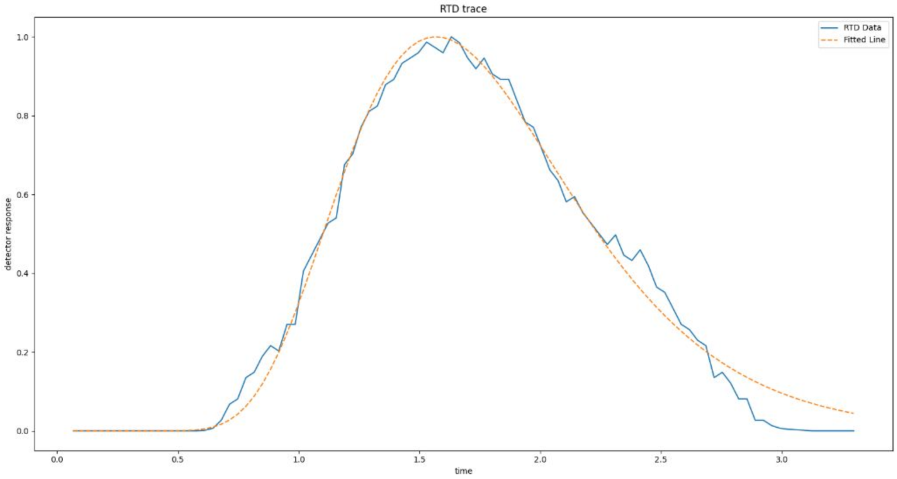

Fitted RTD of Vaportec

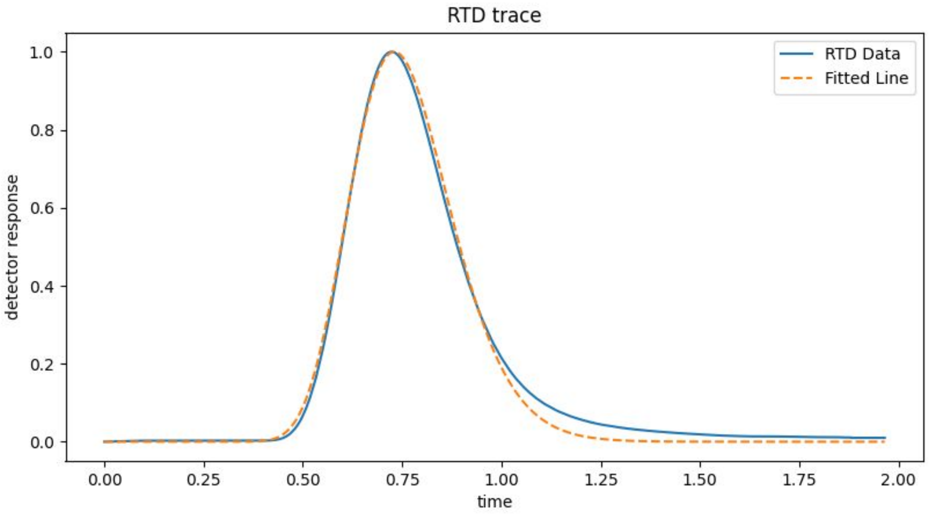

Fitted RTD of SABRe

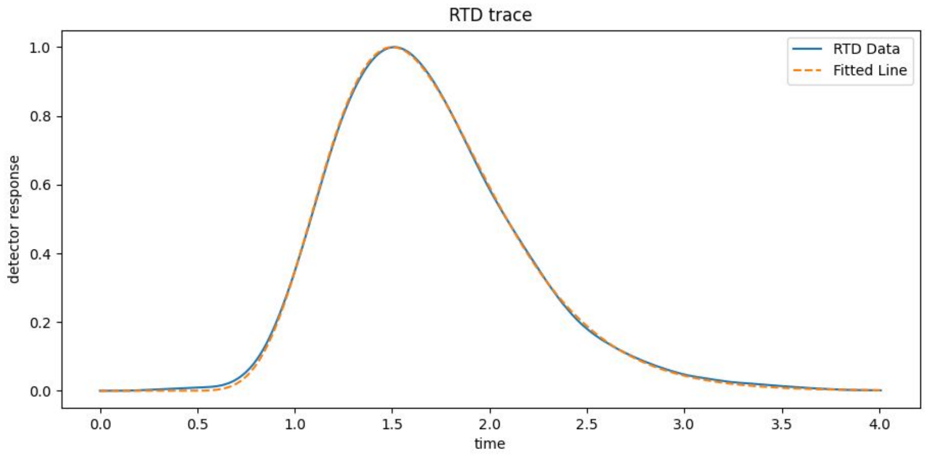

2. CCTP results

2.1 Stability test for three systems

2.1.1 AFR

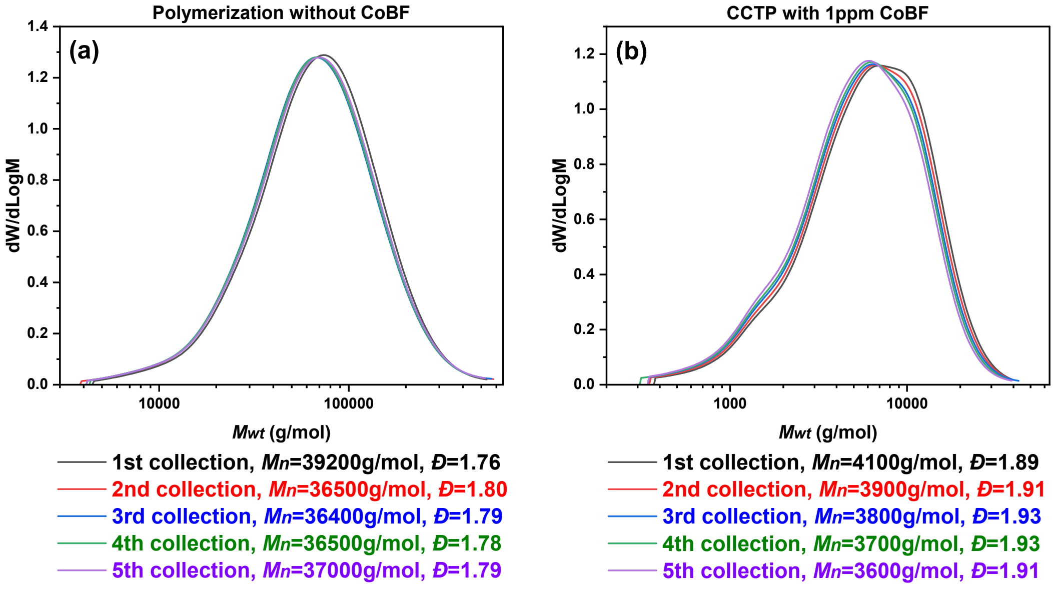

SF 1. GPC traces of polymer product for polymerization with and without CoBF in AFR under 90 ℃ for 5 mins, Samples were collected each 5 min after average residence time.

2.1.2 Vaportec

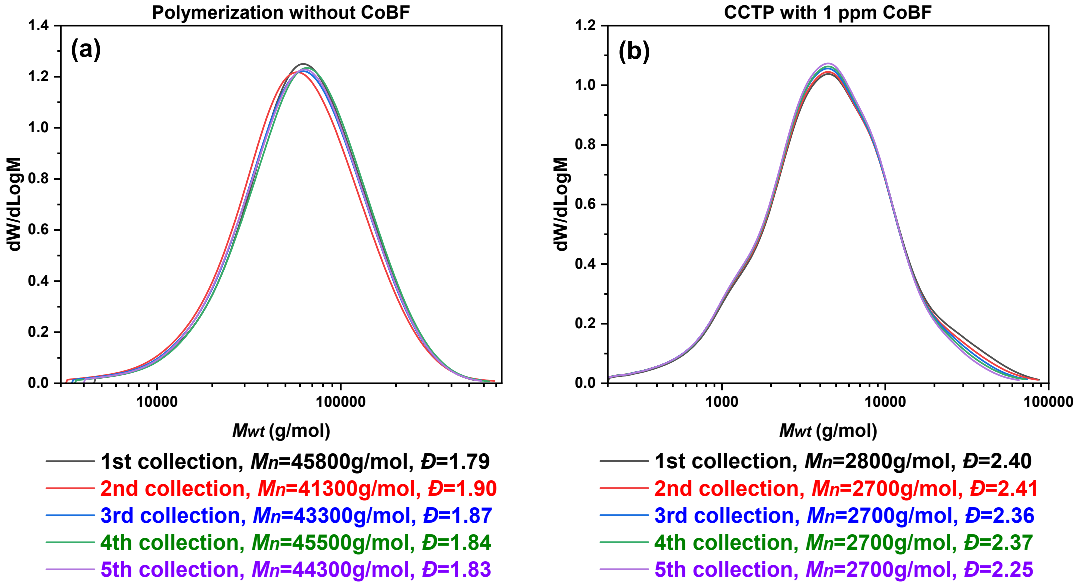

SF2. GPC traces of polymer product for polymerization with and without CoBF in Vaportec under 70 ℃ for 20 mins, Samples were collected each 5 min after average residence time.

2.1.3. SABRe

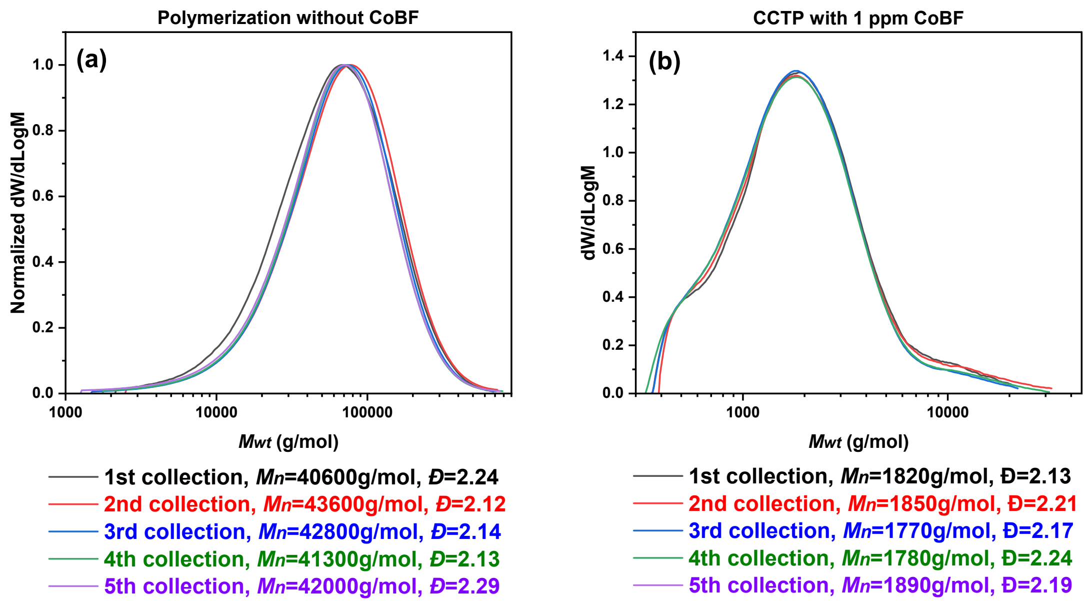

SF 3. GPC traces of polymer product for polymerization with and without CoBF in SABRe under 70 ℃, 100 rpm for 20 mins, Samples were collected each 5 min after average residence time.

2.2 CCTP results for different systems under varied temperatures and monomer.

2.2.1 Stirring rate

Table 1. Stirring rate impact on the polymer products' properties and CoBF efficiency.

Stirring rate (rpm)	CoBF (ppm)	*Mw* (*g/mol*)	*Đ*	Conv. (%)	C_s
100	0	80800	2.2	5.2	42200
	1	3500	2.2	4.9	
	2	2100	2.6	4.8	
	3	1400	2.5	4.6	
	4	1200	2.4	3.4	
	5	900	2.6	3.4	
200	0	40800	2.0	8.9	46800
	1	3200	2.3	5.9	
	2	1600	2.1	5.3	
	3	1300	2.7	5.1	
	4	900	1.9	4.9	
	5	800	2.2	4.6	
300	0	18300	2.7	21.9	49600
	1	2800	2.6	14.9	
	2	1300	2.2	13.3	
	3	1000	1.9	13.2	
	4	800	2.2	12.9	
	5	700	2.7	11.9	

2.2.1 Temperature dependence

Batch

Table 2. Temperature influence on the polymer products' properties and CoBF efficiency in batch.

T (℃)	CoBF (ppm)	M_w (g/mol)	Đ	Conv. (%)	C_s
70	0	110600	2.3	9.1	58700
	1	4600	1.6	4.7	
	2	1600	2.7	3.4	
	3	1100	2.2	3.2	
	4	800	2.0	3.6	
	5	700	1.8	4.2	
80	0	70200	2.1	18.4	47400
	1	8500	1.9	11.7	
	2	3300	2.9	8.6	
	3	2100	2.5	7.7	
	4	1400	2.3	7.4	
	5	1100	2.1	6.7	
90	0	38200	2.1	32.8	35300
	1	6500	1.9	26.1	
	2	3100	2.6	22.5	
	3	1900	2.5	19.2	
	4	1600	2.5	16.9	
	5	1100	2.4	13.3	

SABRe

Table 3. Temperature influence on the polymer products' properties and CoBF efficiency in SABRe.

T (ºC)	CoBF (ppm)	M_w (g/mol)	$Đ$	Conv.[b] (%)	C_s
70	0	80800	2.2	5.2	42200
	1	3500	2.2	4.9	
	2	2100	2.6	4.8	
	3	1400	2.5	4.6	
	4	1200	2.4	3.4	
	5	900	2.6	3.4	
80	0	54800	2.1	13.0	48400
	1	2500	2.2	10.7	
	2	1500	2.4	10.4	
	3	1200	2.5	9.9	
	4	900	2.0	9.1	
	5	800	2.6	7.0	

AFR

Table 4. Temperature influence on the polymer products' properties and CoBF efficiency in AFR.

T (ºC)	CoBF (ppm)	Mw (g/mol)	Đ	Conv. (%)	Cs
70	0	110000	1.9	5.1	21600
	1	10800	2.4	5.0	
	2	6400	2.3	4.7	
	3	4000	2.4	4.5	
	4	2400	2.2	3.6	
	5	1800	2.1	3.4	
80	0	125000	1.7	6.5	24800
	1	8500	1.9	6.0	
	2	5300	2.1	5.9	
	3	3100	2.3	5.3	
	4	2100	2.1	4.6	
	5	1600	2.0	4.8	
90	0	68000	2.1	7.3	24100
	1	7200	2.8	6.6	
	2	3600	2.4	5.7	
	3	2500	2.5	5.4	
	4	1800	2.0	5.6	
	5	1600	2.6	4.7	

Vaportec

Table 5. Temperature influence on the polymer products' properties and CoBF efficiency in Vaportec.

T (ºC)	CoBF (ppm)	M_w (g/mol)	$Đ$	Conv. (%)	C_s
70	0	92400	2.0	6.8	47600
	1	4600	2.2	6.0	
	2	1900	2.6	5.2	
	3	1300	2.5	4.0	
	4	1100	2.4	2.9	
	5	800	2.2	3.0	
80	0	57400	2.0	15.6	42000
	1	4200	2.6	13.0	
	2	2300	2.6	12.7	
	3	1600	2.5	12.2	
	4	1200	2.5	12.0	
	5	900	2.4	11.0	
90	0	39100	2.0	26.6	42800
	1	4200	2.6	23.5	
	2	2500	2.6	22.2	
	3	1700	2.5	22.0	
	4	1300	2.5	21.1	
	5	900	2.3	19.4	

2.2.2 Different Methacrylates

Batch

Table 6. Monomer influence on the polymer products' properties and CoBF efficiency in batch.

Monomer	CoBF (ppm)	*Mw* (g/mol)	*Đ*	Conv. (%)	C_s
MMA	0	110600	2.3	9.1	58700
	1	4600	1.6	4.7	
	2	1600	2.7	3.4	
	3	1100	2.2	3.2	
	4	800	2.0	3.6	
	5	700	1.8	4.2	
BMA	0	127400	3.96	12.8	53800
	1	8900	1.8	5.2	
	2	3400	2.1	5.0	
	3	1900	2.2	4.4	
	4	1500	1.8	3.5	
	5	1000	1.7	.2.9	
BzMA	0	107400	4.9	18.3	22100
	1	24000	2.1	13.6	
	2	7400	2.3	10.1	
	3	4300	2.3	7.5	
	4	4100	2.4	6.2	
	5	3300	2.3	5.8	

SABRe

Table 7. Monomer influence on the polymer products' properties and CoBF efficiency in SABRe.

Monomer	CoBF (ppm)	*Mw* (g/mol)	Đ	Conv. (%)	C_s
MMA	0	80800	2.2	5.2	42200
	1	3500	2.2	4.9	
	2	2100	2.6	4.8	
	3	1400	2.5	4.6	
	4	1200	2.4	3.4	
	5	900	2.6	3.4	
BMA	0	79900	2.0	7.8	30600
	1	7500	2.1	6.1	
	2	3100	2.3	6.0	
	3	2100	2.2	5.4	
	4	1700	2.1	4.7	
	5	1600	2.1	3.3	
BzMA	0	66960	2.9	6.1	27000
	1	9400	2.1	4.8	
	2	3200	2.4	5.3	
	3	2500	2.2	3.7	
	4	1800	2.2	3.8	
	5	1600	2.0	3.1	

AFR

Table 8. Monomer influence on the polymer products' properties and CoBF efficiency in AFR.

Monomer	CoBF (ppm)	*Mw* (g/mol)	*Đ*	Conv. (%)	C_s
MMA	0	110000	1.9	5.1	21600
	1	10800	2.4	5.0	
	2	6400	2.3	4.7	
	3	4000	2.4	4.5	
	4	2400	2.2	3.6	
	5	1800	2.1	3.4	
BMA	0	141000	2.5	5.8	18700
	1	18300	2.2	5.1	
	2	9900	2.6	5.4	
	3	4200	2.4	5.9	
	4	2600	2.0	5.6	
	5	2200	2.0	4.7	
BzMA	0	138000	2.9	6.1	16300
	1	13600	2.9	5.4	
	2	7000	2.8	5.3	
	3	4100	2.8	5.1	
	4	2700	2.7	4.8	
	5	1700	2.9	4.1	

Vaportec

Table 9. Monomer influence on the polymer products' properties and CoBF efficiency in Vaportec.

Monomer	CoBF (ppm)	*Mw* (g/mol)	Đ	Conv. (%)	C_s
MMA	0	92400	2.0	6.8	47600
	1	4600	2.2	6.0	
	2	1900	2.6	5.2	
	3	1300	2.5	4.0	
	4	1100	2.4	2.9	
	5	800	2.2	3.0	
BMA	0	79300	2.8	9.6	28800
	1	8700	2.3	6.5	
	2	4500	2.1	5.1	
	3	3200	2.0	4.8	
	4	2500	1.9	3.7	
	5	1900	1.9	3.9	
BzMA	0	100500	3.4	15.3	19500
	1	22500	3.2	9.5	
	2	8300	2.4	7.7	
	3	5900	2.5	6.1	
	4	4400	2.3	5.9	
	5	3600	2.5	6.0	

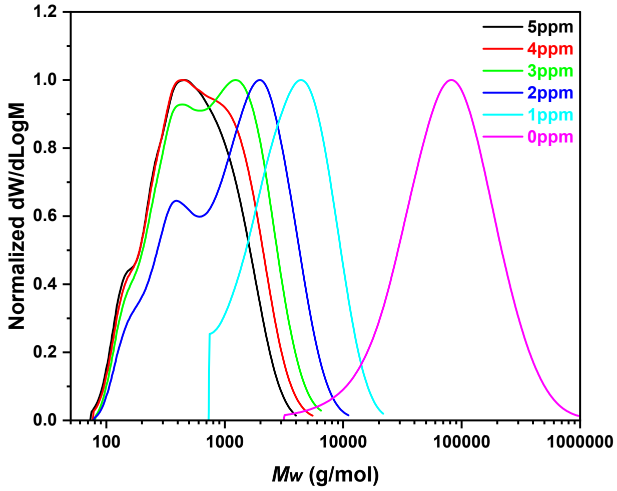

SF4. GPC traces of CCTP with MMA in 3 ml vials under 70 °C, 200 stirring rate, 20 minutes.

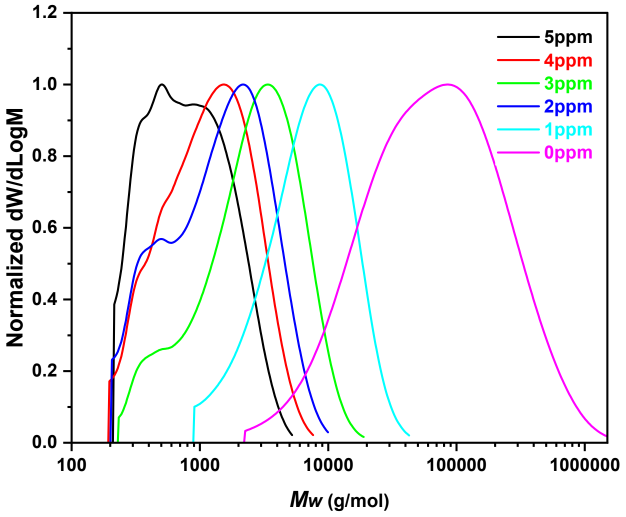

SF5. GPC traces of CCTP with n-BMA in 3 ml vials under 70 ^{o}C, 200 stirring rate, 20 minutes.

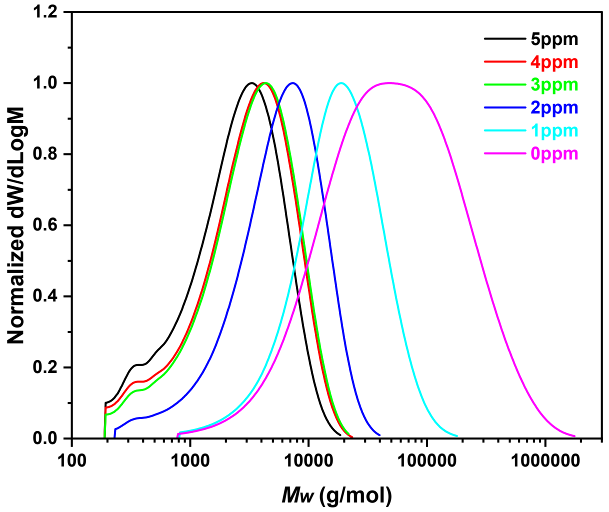

SF6. GPC traces of CCTP with BzMA in 3 ml vials under 70 ^{o}C, 200 stirring rate, 20 minutes.

Vapourtec

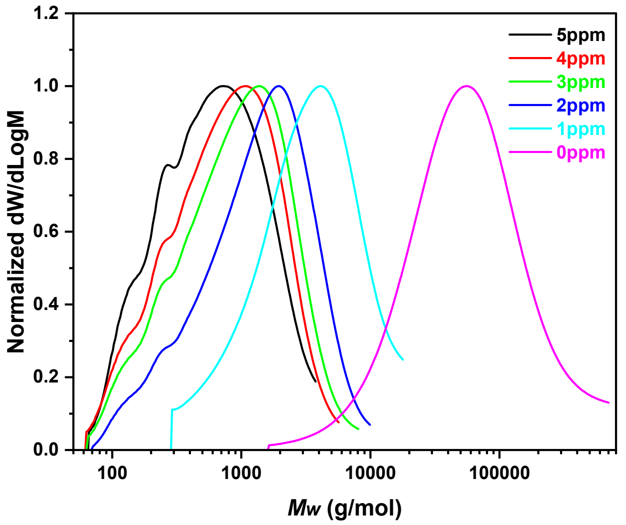

SF7. GPC traces of CCTP with MMA in a 25 tubular coil reactor under 70 °C, 20 minutes.

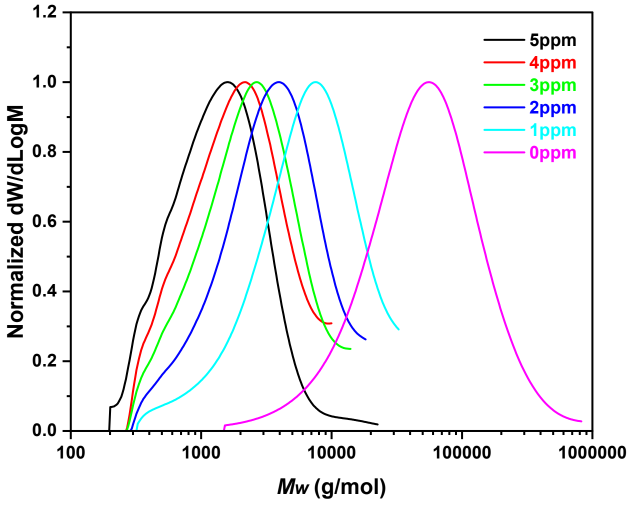

SF8. GPC traces of CCTP with n-BMA in a 25 tubular coil reactor under 70 °C, 20 minutes.

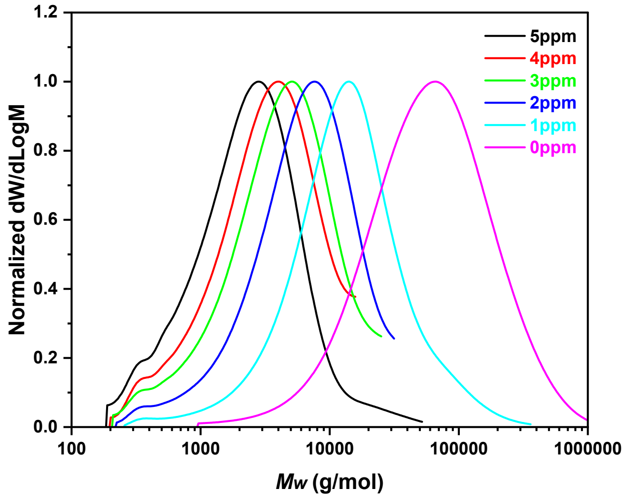

SF9. GPC traces of CCTP with BzMA in a 25 tubular coil reactor under 70 °C, 20 minutes.

<u>SABRe</u>

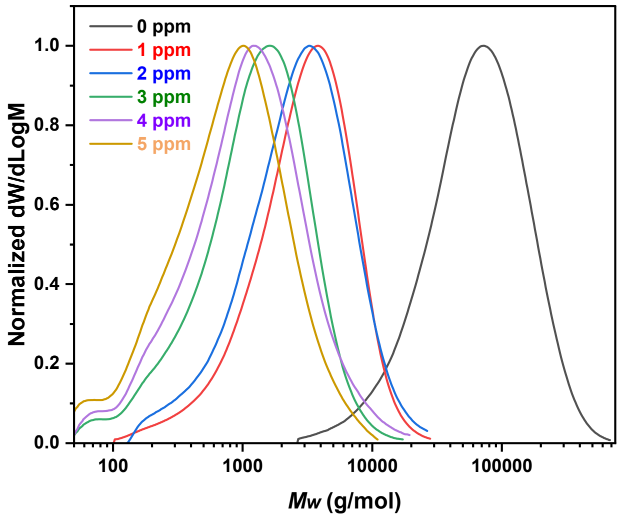

SF10. GPC traces of CCTP with MMA in SABRe reactor under 70 °C, 20 minutes, 100 rpm.

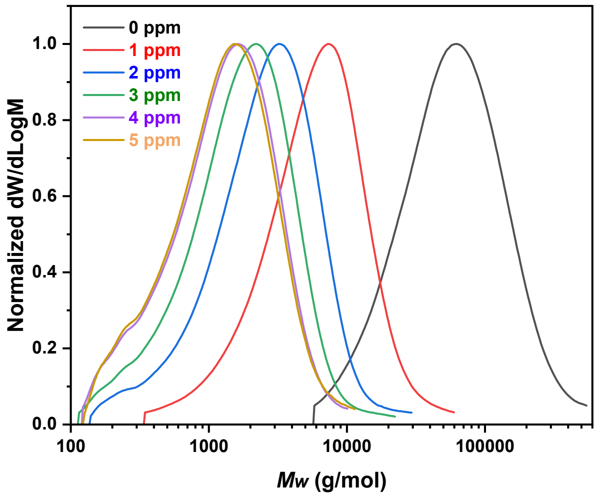

SF11. GPC traces of CCTP with n-BMA in SABRe reactor under 70 °C, 20 minutes, 100 rpm.

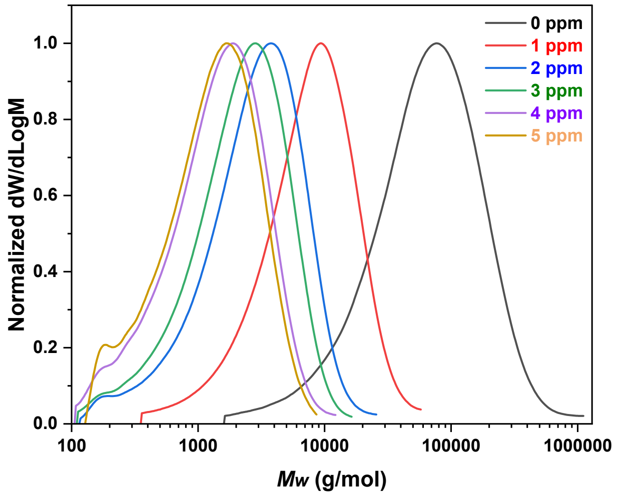

SF12. GPC traces of CCTP with BzMA in SABRe reactor under 70 °C, 20 minutes, 100 rpm.

Corning

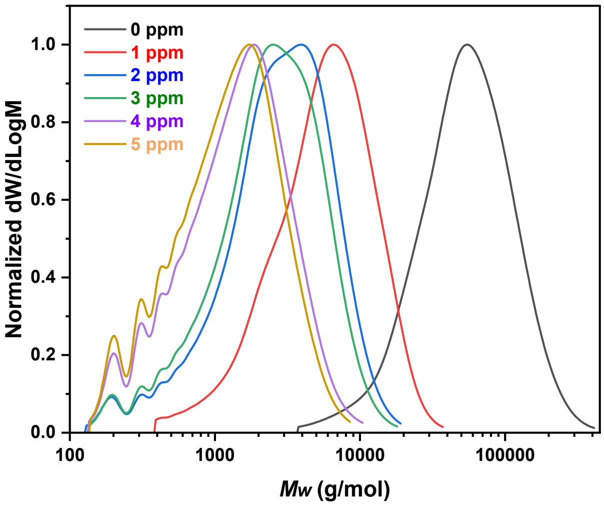

SF13. GPC traces of CCTP with MMA in Corning reactor under 90 ^{o}C, 5 minutes.

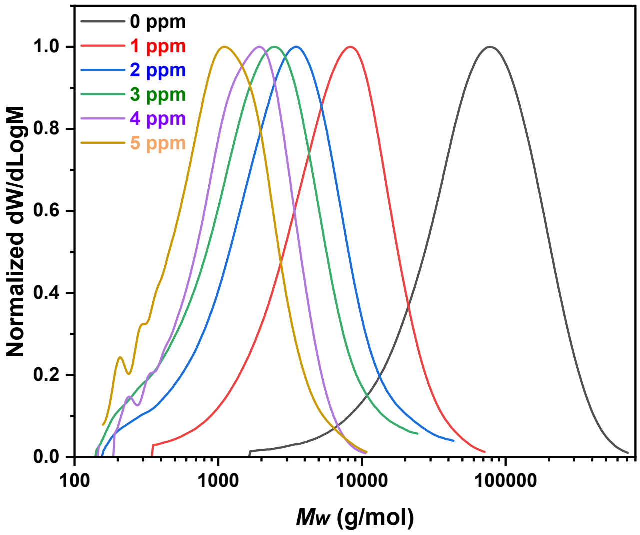

SF14. GPC traces of CCTP with n-BMA in Corning reactor under 90 ^{o}C, 5 minutes.

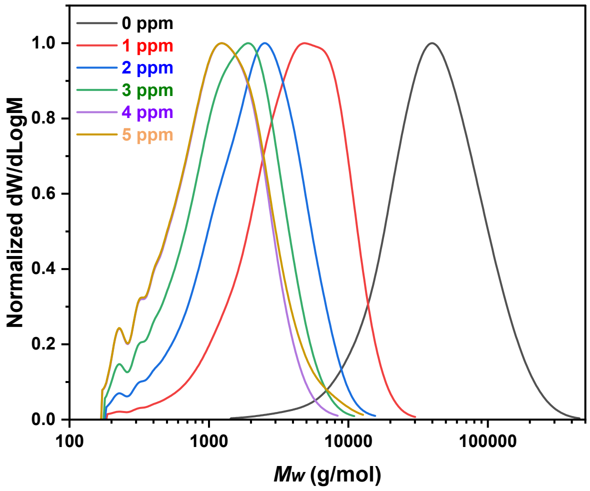

SF15. GPC traces of CCTP with BzMA in Corning reactor under 90 °C, 5 minutes.
